# RETRACTION**: lncRNA BZRAP1‐AS1 Alleviates Rheumatoid Arthritis by Regulating miR‐1286/COL5A2 Axis**

**DOI:** 10.1002/iid3.70204

**Published:** 2025-05-28

## Abstract

**RETRACTION:** J. Zhu, S. Tu and Q. Qu, “lncRNA BZRAP1‐AS1 Alleviates Rheumatoid Arthritis by Regulating miR‐1286/COL5A2 Axis,” *Immunity, Inflammation and Disease* 10, no. 2 (2022): 163‐174, https://doi.org/10.1002/iid3.558.

The above article, published online on 11 November 2021 in Wiley Online Library (wileyonlinelibrary.com), has been retracted by agreement between the journal Editor‐in‐Chief, Marc Veldhoen; and John Wiley & Sons, Ltd. The retraction has been agreed upon due to inconsistencies and flaws identified in this article that affect the validity of the conclusions. The authors did not respond to address the concerns raised and did not provide their original data. The editors consider the results and conclusions reported in this article unreliable. The authors were informed of the retraction.

**RETRACTION:** J. Zhu, S. Tu and Q. Qu, “lncRNA BZRAP1‐AS1 Alleviates Rheumatoid Arthritis by Regulating miR‐1286/COL5A2 Axis,” *Immunity, Inflammation and Disease* 10, no. 2 (2022): 163‐174, https://doi.org/10.1002/iid3.558.

The above article, published online on 11 November 2021 in Wiley Online Library (wileyonlinelibrary.com), has been retracted by agreement between the journal Editor‐in‐Chief, Marc Veldhoen; and John Wiley & Sons, Ltd. The retraction has been agreed upon due to inconsistencies and flaws identified in this article that affect the validity of the conclusions. The authors did not respond to address the concerns raised and did not provide their original data. The editors consider the results and conclusions reported in this article unreliable. The authors were informed of the retraction.

